# Dental tissue remineralization by bioactive calcium phosphate nanoparticles formulations

**DOI:** 10.1038/s41598-022-09787-5

**Published:** 2022-04-09

**Authors:** Andrei Cristian Ionescu, Lorenzo Degli Esposti, Michele Iafisco, Eugenio Brambilla

**Affiliations:** 1 Oral Microbiology and Biomaterials Laboratory, Department of Biomedical, Surgical, and Dental Sciences, University of Milan, Via Pascal, 36, 20133 Milan, Italy; 2 Institute of Science and Technology for Ceramics (ISTEC), National Research Council (CNR), Via Granarolo, 64, 48018 Faenza, Italy

**Keywords:** Biological techniques, Chemical biology, Medical research, Materials science, Nanoscience and technology

## Abstract

Recent health care products are based on formulations claimed to provide enamel remineralization and dentinal tubules occlusion through calcium-phosphate bioactive nanocompounds (ion-doped hydroxyapatite and precursor, amorphous calcium phosphate nanoparticles). This study aimed to characterize, test, and compare for the first time the structure and performance of a representative, market-available sample of remineralizing toothpastes and topical mousses. Formulations were characterized to determine their composition and investigate the presence of bioactive compounds and doping elements. A conventional fluoride-containing toothpaste was used as reference. The enamel remineralization and efficacy of dentinal tubules occlusion by tested formulations were investigated ex vivo on human hard tissues. All formulations containing Ca-P bioactive nanocompounds showed remineralizing ability by epitaxial growth of a layer showing the morphology and composition of human hydroxyapatite. Such layers also embedded nanosilica clusters. The presence of doping elements or casein phosphopeptide seemed essential to allow such performances, especially when hydroxyapatite and amorphous calcium phosphate compounds were doped with small amounts of CO_3_^2−^, F^−^, Mg^2+^, and Sr^2+^. Topical mousse formulations showed a higher tubules occlusion capability than toothpastes, independently from their composition. Therefore, all tested formulations could be useful in restoring tooth structures in a biomimetic way, contrasting dental demineralization processes leading to caries.

## Introduction

Enamel demineralization is the primary mechanism involved in the etiopathogenesis of dental caries and erosion^1^. When the oral microenvironment reaches low pH values (i. e., lower than the critical pH of dental surfaces), the demineralization process is triggered. This phenomenon represents the outcome of very complex biological and chemical interactions taking place at the interface between the external oral environment, the biofilm colonizing hard tissues, and the dental hard tissues themselves^2,3^. Hydroxyapatite (HA) is the main component of dental enamel, which is soluble in acidic solutions and has a critical pH of about 5.5. Dentine, having a lower mineral content, shows an even higher critical pH (about 6.2) due to its lower crystallinity. Below these pH values, an ionic imbalance progressively causes the loss of ionic species from the hard tissues and the consequent dissolution of the tissue itself^4^. Several factors can cause a pH drop to values much lower than 5.5 and trigger dental demineralization, such as the fermentative activity of dental biofilms, the intake of acidic food or beverages, and gastroesophageal reflux^5,6^. 

In physiological conditions, salivary ion content and buffering capacity can compensate for the demineralization process, thus creating a delicate equilibrium where demineralization and remineralization phases occur, resulting in an even ionic balance^7^. Changes in the oral microenvironment, such as a significant carbohydrate intake, can impair this equilibrium and favor demineralization^3^. Therefore, an external supply of calcium and phosphate ions was proposed to shift the equilibrium towards remineralization^8^.

Demineralization is a reversible process. When the microenvironment reaches pH values higher than 7.0 and there is an availability of calcium and phosphate ions, remineralization can occur. Such ions precipitate, covering the dental surfaces with an amorphous mineral layer. Under defined conditions, however, this layer can act as a precursor to form a more organized mineral structure^8–10^. This phenomenon is called epitaxial growth. It was demonstrated that impaired enamel hydroxyapatite prisms could guide epitaxial growth, starting a biomineralization process to restore demineralized hard tissues^11,12^. Toothpastes and, more recently, topical formulations (mousses) are an effective and reliable way to deliver active principles to dental hard tissues, notwithstanding differences in their application protocols. Indeed, topical formulations are designed to be applied using universal or customized trays that allow prolonged contact of the formulation with the tooth tissues, thus providing a relatively high delivery of ionic species to hard tissues. Therefore, the inclusion of compounds with remineralizing capabilities in toothpaste and topical formulations has become a common practice nowadays^13,14^.

Among these compounds, fluoride is still considered the gold standard in inhibiting demineralizing processes^15,16^. This ion has a dual anti-caries activity: (i) it directly inhibits the metabolic pathways of microbial cells in the overlying biofilm and (ii) the accumulation of fluoride at the enamel surface leads to the precipitation of calcium fluoride-like deposits, which act as a reservoir and gradually release fluoride during conditions of decreased pH values^17^. The released fluoride prevents enamel demineralization and reduces caries susceptibility by incorporating fluoride into enamel hydroxyapatite via filling or displacing the hydroxyl vacancies, therefore stabilizing the crystal structure forming fluorapatite and lowering the solubility product constant (Ksp). Consequently, fluoride-treated dental tissues show increased resistance to demineralization. Also, biofilms’ acidogenicity decreases.

Synthetic HA and other calcium phosphate-based systems such as amorphous calcium phosphate (ACP) particles and especially nanoparticles promoted remineralization processes^18,19^. Furthermore, the doping of HA with ions, such as CO_3_^2−^, Zn^2+^, F^−^, Mg^2+^, and Sr^2+^, has been shown to modulate the crystals’ nucleation, growth, orientation, and solubility. In fact, biogenic dental HA contains small amounts of CO_3_^2−^, Mg^2+^, and Sr^2+^, having the same effects^8,19^. ACP was shown to release significantly higher calcium and phosphate ions than other CaP phases, including HAp, as ACP is the most soluble CaP phase. However, ACP is a metastable material that is quickly converted to more stable CaP phases when in contact with water or moisture and represents the transient phase of natural HAp formation^20^. Several formulations have been proposed to stabilize ACP for enamel remineralization, among which the most common are casein phosphopeptides (CPP)^21^ and, more recently, citrate (Cit-ACP)^8^.

Both HAp and ACP found successful application as active ingredients for remineralizing toothpastes and remineralizing topical formulation (mousses). Several products that claim a remineralizing effect containing these calcium phosphates phases in the form of stabilized nanoparticles are sold worldwide as over-the-counter products.

This work aimed to compare the chemical and physical composition and in vitro bioremineralization activity of five commercially-available toothpaste and topical formulations containing ion-substituted HA, Cit-ACP, or CPP-ACP with a conventional fluoride-containing toothpaste, kept as a reference. Additional aim was to correlate the composition of the tested formulation with their activity.

The null hypothesis was that the remineralization activity of the tested toothpastes is not significantly different from that of a fluoride-containing conventional toothpaste used as control.

## Results

The formulations tested in this study and their ingredients as specified by the manufacturers are illustrated in Table 1.

**Table 1:** Specified ingredients of the tested toothpastes and mousses.

Formulation type	Name (abbrv.)	Company	Specified ingredients
Toothpaste formulation	Biosmalto caries abrasion and erosion (BT)	Curaden Healthcare, Saronno (VA), Italy	Aqua, glycerin, hydrated silica, **fluoride-hydroxyapatite (1450 ppm F), Mg-Sr-carbonate hydroxyapatite conjugated with chitosan**, cellulose gum, xylitol, cocamidopropyl betaine, xantham gum, aroma, acesulfame K, ethylhexylglycerin, phenoxyethanol, sodium benzoate, citric acid
Toothpaste formulation	Biorepair Plus (RT)	Coswell, Funo (BO), Italy	Aqua, **zinc carbonate hydroxyapatite**, glycerin, sorbitol, hydrated silica, silica, aroma, cellulose gum, tetrapotassium pyrophosphate, sodium myristoyl sarcosinate, sodium methyl cocoyl taurate, sodium saccharin, citric acid, phenoxyethanol, benzyl alcohol, sodium benzoate
Toothpaste formulation (reference)	Colgate triple action (CT)	Colgate-Palmolive, Guilford, UK	Aqua, sorbitol, hydrated silica, glycerin, sodium lauryl sulfate, PEG-12, peppermint oil, spearmint oil, cellulose gum, **NaF (1450 ppm F)**, sodium saccharin, CI 74,160, CI 74,260, TiO2
Topical formulation (mousse)	Biosmalto Caries Protection (BM)	Curaden	Glycerin, PEG-8, silica, strontium acetate, **calcium phosphate carbonate citrate fluoride**, hydroxypropyl cellulose, xylitol, acrylates/C10-30 alkyl acrylate crosspolymer, Peg-40 hydrogenated castor oil, sodium hyaluronate, potassium acesulfame, P-anisic acid, aroma, sodium hydroxide
Topical formulation (mousse)	Biorepair desensitizer enamel repair (RM)	Coswell	Aqua, **zinc carbonate hydroxyapatite**, hydrated silica, silica, sodium myristoyl sarcosinate, sodium methyl cocoyl taurate, cellulose gum, sodium bicarbonate, aroma, sodium saccharin, phenoxyethanol, benzyl alcohol, sodium benzoate, citric acid, menthol
Topical formulation (mousse)	MI Paste Plus (MM)	GC Corporation, Tokyo, Japan	Aqua**, Recaldent (10% CPP-ACP), fluoride (900 ppm F)**, glycerol, d-sorbitol, CMC-Na, propylene glycol, silicon dioxide, titanium dioxide, xylitol, phosphoric acid, flavoring, sodium saccharin, ethyl p-hydroxybenzoate, propyl p-hydroxybenzoate, butyl p-hydroxybenzoate

The remineralizing ingredients, including the sources of fluoride ions, are highlighted in bold.

### Structural characterization

All toothpastes contain 15–20 wt.% water-insoluble inorganic matter, 20–40 wt.% water-soluble matter, and 40–50 wt.% water (Table 2).

**Table 2:** Composition of each tested formulation resulting from freeze-drying, water-washing and acid-washing procedures.

Formulation	Water (wt%)	Water-soluble fraction (wt%)	Water-insoluble fraction (wt%)	
			Calcium phosphate (wt%)	Silica (wt%)
BT	52 ± 7^a^	33 ± 3^a^	5.0 ± 0.5^a^	10 ± 1^a^
RT	55 ± 1	22 ± 1	15 ± 1	9 ± 1
CT (reference)	41 ± 1	42 ± 2	0 ± 0	17 ± 1
BM	10 ± 1	78 ± 1	2.0 ± 0.5	10 ± 1
RM	63 ± 1	11 ± 1	20 ± 1	6 ± 1
MM	54 ± 1	42 ± 3	1.0 ± 0.1	3.0 ± 0.5

Superscript (a), data published in Degli Esposti et al*.*^18^. Mean wt% for each group is shown ± 1 standard deviation.

Among the toothpastes, only BT and RT have an acid-soluble inorganic phase, while CT contains acid-insoluble SiO_2_ and TiO_2_, in agreement with the ingredients list. BT and RT have a different weight ratio between CaP and SiO_2_, namely RT contains 15 wt.% of CaP and 9 wt.% of SiO_2_ (CaP / SiO_2_ = 1.7) while BT presents 5 wt.% of CaP and 10 wt.% of SiO_2_ (CaP / SiO_2_ = 0.5).

Considering topical formulations, RM has the highest water content, the lowest water-soluble matter content, and it has a higher amount of CaP than SiO_2_. On the contrary, BM contains the lowest amount of water and the highest content of water-soluble matter. BM, differently from RM, contains more SiO_2_ than CaP. MM has a peculiar composition made by a low amount of water-insoluble components (ca. 1 wt.% CaP and 3 wt.% SiO_2_) and ca. 95% of weight made by water and water-soluble ingredients.

**Figure 1:**
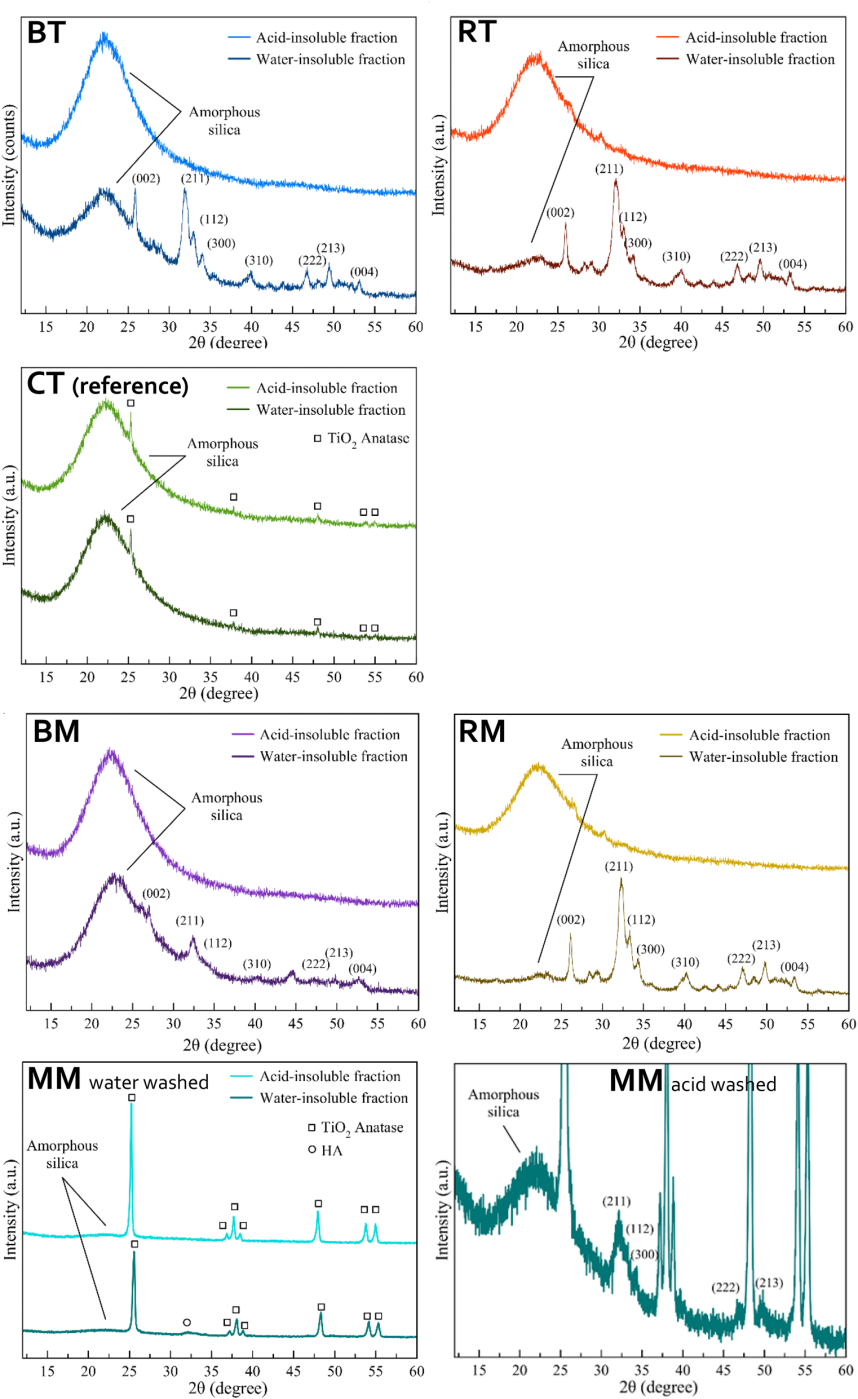
PXRD patterns of the tested formulations.

The water-insoluble component of the products before and after washing with the acid solution was analyzed by PXRD (Fig. 1). The PXRD patterns of the water-insoluble phase of BT and RTs have similar diffraction patterns (Fig. 1), showing a broad band centered at 23° that corresponds to amorphous SiO_2_ and several diffraction peaks that were indexed as HA (PDF card file 09-0432).

The intensity of the SiO_2_ band in comparison to the intensity of HA peaks is higher in BT than in RT, in agreement with the higher SiO_2_ content evaluated by compositional analysis. The HA peaks are broadened in both toothpastes, indicating a poor crystallinity and a high material similarity to biogenic HA. After the acidic washing of both toothpastes, the HA peaks disappear, which confirms that the process successfully removes the CaP components. In the PXRD patterns of the reference CT, the band of amorphous SiO_2_ is present, together with weak diffraction peaks that were indexed as anatase (TiO_2_, PDF card file 21-1272), and there are no changes after acidic washing, confirming that no CaP is present in the control product.

The PXRD pattern of the water-insoluble fraction of BM shows amorphous SiO_2_ and HA peaks, with the latter being relatively low and broad. Also, the PXRD pattern of water-insoluble RM fraction presents SiO_2_ and HA peaks, but the HA peaks are more intense and resolved than BM. This finding suggests that RM has a higher HA content in agreement with compositional analysis, and its HA has a higher crystallinity. Anatase diffraction peaks dominate the PXRD pattern of the water-insoluble phase of MM, while amorphous SiO_2_ and HA signals were also observed (Fig. 1, MM water-washed and acid-washed). As for BM, the washing process has likely converted the ACP contained as CPP-ACP into HA. For all topical formulations, the HA peaks are not present in the PXRD patterns of the acid-insoluble fractions, confirming the removal of CaP thanks to the acidic washing.

**Figure 2:**
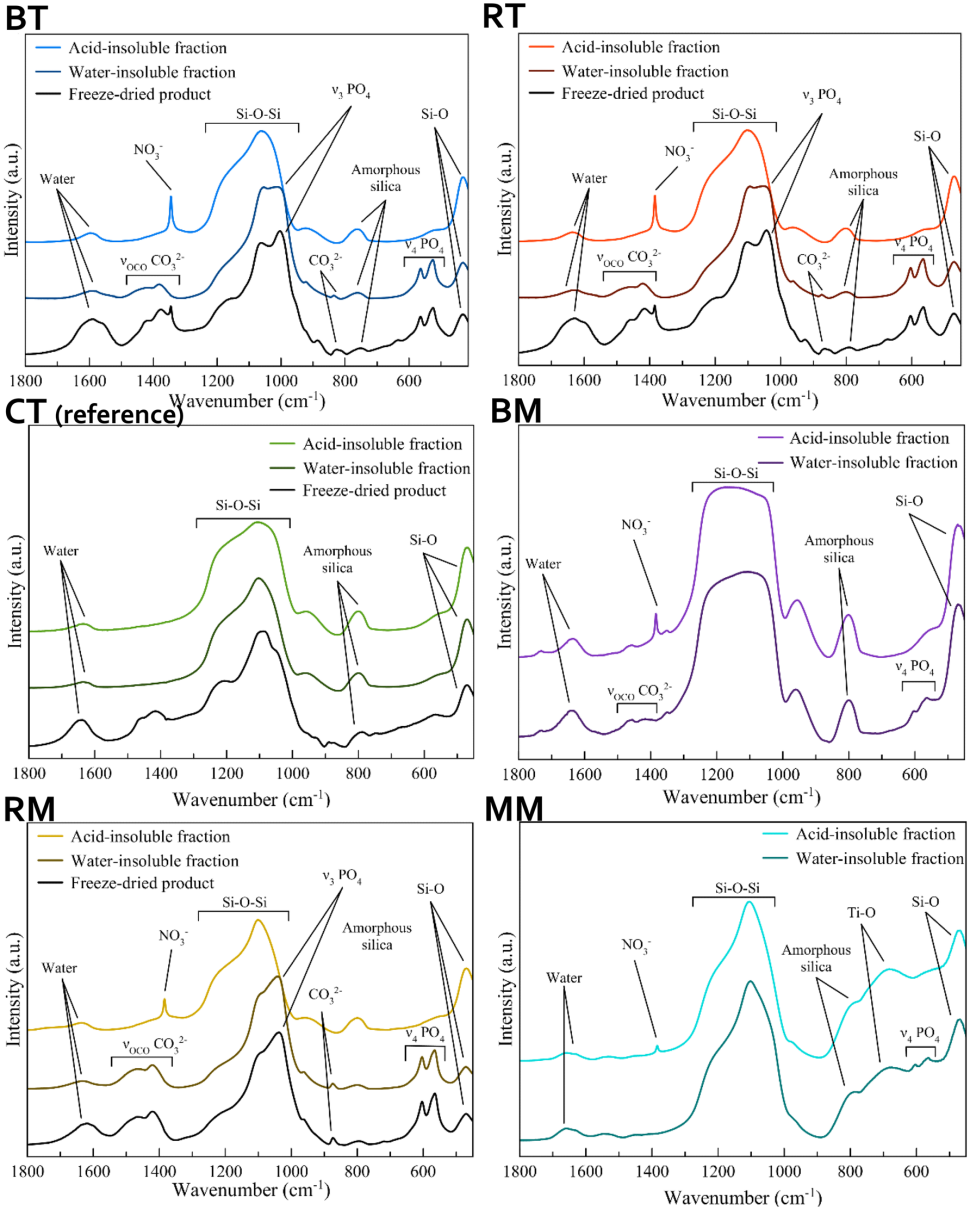
FT-IR spectra of the tested formulations.

The products were also investigated by FT-IR spectroscopy. The FT-IR spectra of the freeze-dried whole formulations, the water-insoluble fractions, and the acid-insoluble fractions were collected (Fig. 2). In the case of BM and MM, the freeze-drying did not yield a dry product but formed a thick paste, and therefore for these samples, the FT-IR of the whole product could not be collected. In general, the spectra of the whole products are of complex interpretation due to the superposition of vibrational bands of SiO_2_, CaP, and organic molecules. Some components were removed with the aqueous and acidic washings, allowing to associate the IR peaks of each phase better.

The FT-IR spectra of the water-insoluble fraction of BT and RT present the same bands. The main bands are due to SiO_2_ and fall at ca. 455 cm^−1^ and 1055 cm^−1^, associated with the Si–O rocking and the Si–O–Si siloxane vibration, respectively. In addition, a weak band present at ca. 800 cm^−1^ is representative of amorphous SiO_2_. These samples present two bands at 603 and 565 cm^−1^ due to the bending vibration of HA phosphate groups (ν_4_PO_4_ mode). The Si–O–Si bands of these two toothpastes present a shoulder at 1040 cm^−1^ that is attributed to the ν_3_PO_4_ band (phosphate stretching vibration) of HA. Also, the intensity ratio between Si–O–Si and ν_4_PO_4_ bands suggests that RT has a higher HA to SiO_2_ ratio than BT. In the BT and RT spectra, there are three weak bands at ca. 870 cm^−1^, 1415 cm^−1^, and 1460 cm^−1^ belonging to the bending and stretching vibrations of CO_3_^2−^ groups that substitute the phosphate groups of HA (carbonate substitution type B). Such a finding confirms the carbonate doping of HA as reported in the ingredients list of the toothpastes (Table 1). In the acid-treated BT and RT spectra, the phosphate and carbonate bands are no more present, confirming that the acidic washing has removed the HA phase and its carbonate doping. A new peak at 1385 cm^−1^ associated with ν_3_NO_3_ vibration of nitrate ions is present in the spectra of all acid-treated samples that can be attributed to the nitrate ions adsorbed on silica after HNO_3_ treatment.

The FT-IR spectrum of the acid-insoluble fraction of the reference CT only shows the bands associated with silica, in agreement with PXRD analysis, and is identical to the spectrum of the water-insoluble fraction. The FT-IR spectrum of the water-insoluble fraction of BM shows the bands associated with SiO_2_, PO_4_^3−^, and CO_3_^2−^. However, in this formulation, the phosphate bands are weaker than those of SiO_2_, in agreement with the lower CaP content of the product. Furthermore, the ν_4_PO_4_ band is split into two sub-bands at 603 and 565 cm^−1^, this splitting being typical of a crystalline HA. The water-insoluble fraction of RM presents the same FT-IR bands shown in the water-insoluble fraction of BM. However, its PO_4_^3−^ and CO_3_^2−^ bands are more intense than BM, agreeing with the compositional analysis.

Finally, the FT-IR spectrum of the water-insoluble MM fraction (Fig. 2) presents the bands associated with SiO_2_ and PO_4_^3−^, along with a band at ca. 680 cm^−1^ that is due to the O–Ti–O vibration of anatase^28^. As seen with BM, the PO_4_^3−^ band in this spectrum is split, indicating the presence of crystalline HA. As seen in PXRD patterns, the PO_4_^3−^ and CO_3_^2−^ FT-IR bands are not present in the acid-insoluble fractions of the three topical formulations because the acidic washing has removed CaP compounds.

### Compositional analysis

The content of Mg^2+^, Sr^2+^, Zn^2+^, and F^-^ ions in the whole formulations and water-insoluble fractions is displayed in Table 3. The total fluoride content of the products is in good agreement with the declared values of BT and BM (1450 ± 50 ppm) while it was a little low (about -20%) compared to the declared values of the reference (CT, 1450 ± 50 ppm) and MM (900 ± 50 ppm). As reported in the ingredients list, RT and RM do not contain F^-^. BT contains both Mg^2+^ and Sr^2+^ ions in the water-insoluble fraction. Besides, RT contains a limited amount of Mg^2+^and has a high amount of Zn^2+^ in the water-insoluble fraction. RM contains low amounts of Mg^2+^ and a high amount of Zn^2+^. According to the manufacturer, BM contains a very high amount of Sr^2+^ as strontium acetate.

**Table 3:** Mg^2+^, Sr^2+^, Zn^2+^, and F content (ppm) in the whole formulations and the water-insoluble fractions.

Formulation	Phase	Mg (ppm)	Sr (ppm)	Zn (ppm)	F (ppm)
BT	Whole product	200 ± 100	900 ± 100	–	1458 ± 25
	Water-insoluble fraction	2000 ± 100	7200 ± 100	–	–
RT	Whole product	110 ± 50	–	1800 ± 50	–
	Water–insoluble fraction	440 ± 90	–	6600 ± 300	–
CT (reference)	Whole toothpaste	–	–	–	1185 ± 34
	Water–insoluble fraction	–	–	–	–
BM	Whole product	–	12,000 ± 1000	–	1300 ± 16
	Water–insoluble fraction	–	22,000 ± 2000	–	–
RM	Whole product	–	–	1390 ± 10	–
	Water–insoluble fraction	610 ± 90	–	5100 ± 400	–
MM	Whole product	–	–	–	731 ± 33
	Water–insoluble fraction	–	–	–	–

Mg^2+^, Sr^2+^, and Zn^2+^ were quantified by inductively-coupled plasma atomic emission spectrometer (ICP-OES), while F^-^ was quantified by the ion-selective electrode (ISE) method. Mean ppm for each group is shown ± 1 standard deviation.

The surface elemental composition of the formulations’ dry mass as obtained using energy-dispersive X-ray spectrometry (EDS) is reported in Table 4. The results are generally in good keeping with the previous analysis. EDS showed a generally similar carbon content in BT, RT, BM, and RM, while in higher amounts in CT and MM. It was shown that the topical formulations (BM, RM) showed higher Ca and P content than their corresponding toothpaste counterparts (BT, RT). An exception was MM that contained surprisingly low Ca and P. The Si content of the formulations correlated with the SiO_2_ content measured in the water- and acid-insoluble fractions.

**Table 4:** Energy-dispersive X-ray spectroscopy (EDS) compositional analysis of the surface layer (≈1 μm) of a 1 g aliquot of each toothpaste tested in the present study.

wt%	BT	RT	CT (reference)	BM	RM	MM
C	25.53	26.08	31.29	24.62	23.96	39.55
O	42.69	43.75	48.83	36.76	34.19	47.37
P	4.93	4.95	0.00	5.82	8.04	1.96
Ca	9.80	12.12	0.00	14.18	20.79	3.92
F	0.18	0.00	0.30	0.20	0.00	0.20
Mg	0.35	0.00	0.00	0.00	0.00	0.00
Zn	0.00	0.27	0.00	0.00	0.22	0.00
Sr	0.93	0.00	0.00	3.79	0.00	0.00
Na	1.38	1.09	0.89	0.73	3.27	1.83
Al	0.32	0.29	0.02	0.15	0.16	0.17
Si	12.12	9.36	17.15	11.52	6.22	2.08
S	0.30	0.25	0.00	0.07	1.88	0.14
Cl	1.08	0.30	0.00	0.57	0.12	0.28
K	0.92	1.56	0.00	1.21	1.01	0.0
Ti	0.00	0.00	1.52	0	0.00	2.39
Ca/P (mol)	1.54	1.89	–	1.88	2.00	1.54

Data are displayed as normalized wt%.

Regarding the doping elements present in small amounts or traces, Mg was correctly detected when in its highest amount (BT), while the analysis did not identify its presence in RT and RM. The amounts of F, Zn, and Sr correlated with the ICP-OES and ISE results. The CT and MM water- and acid-insoluble fractions were confirmed by EDS as containing low amounts of Ti, while all tested formulations contained traces of Al that were twice as high in toothpastes than in topical formulations.

### Remineralization properties of the tested formulations on human enamel

The efficacy of the formulations as enamel remineralizing agents was tested on human enamel specimens that were preliminarily demineralized (acid-etched). After acid etching, negative control samples showed the prismatic structure made by native HA crystals that constitute the primary enamel prisms unit, as etching procedures dissolved the superficial amorphous layer. A demineralization pattern was identified, mainly dissolving the interprismatic mineral phase. The storage solution, only containing phosphate ions, did not remineralize enamel structures.

**Figure 3:**
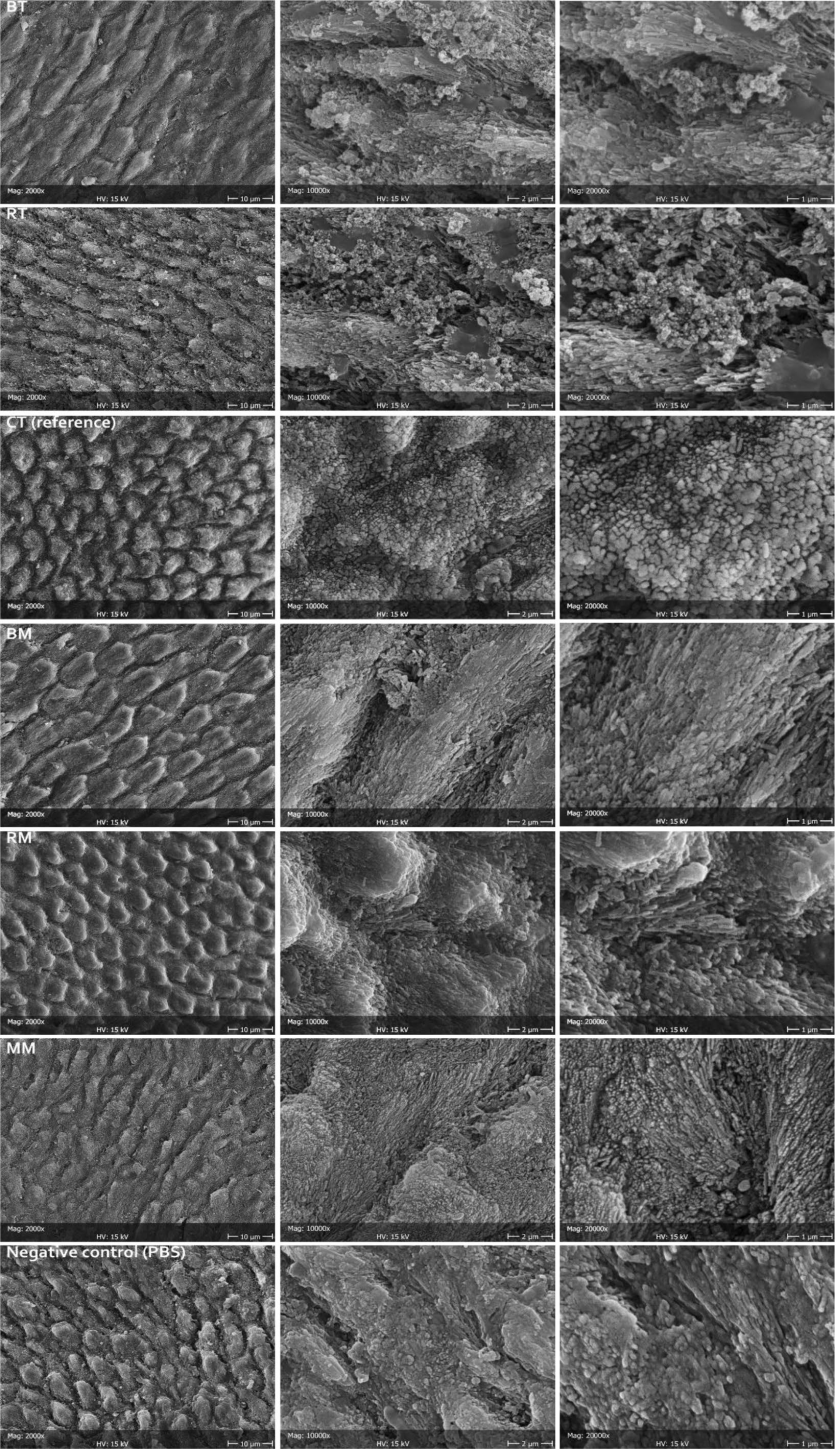
Representative micrographs at different magnifications (2000 × , 10,000 × , and 20,000 ×) of the enamel surface after treatment with the tested and reference formulations, compared to the negative control (demineralized surface stored in PBS).

On the contrary, all tested formulations excepting the reference toothpaste showed remineralization of the demineralized enamel substrate (Fig. 3). Through synoptic comparison of control and treated specimens, it is evident that the repeated application of the formulations for one week has led to the remineralization of the eroded enamel in all tested formulations by depositing a new crystalline phase in direct contact with the pristine one. Epitaxial growth of nanocrystallites was identified that was most pronounced in BT, BM, and MM compared to RT and RM. The fluoride-containing toothpaste (reference CT) showed an even deposition of an amorphous layer on enamel surfaces, which could be due to SiO_2_ deposition from the toothpaste or amorphous calcium fluoride. No signs of epitaxial growth were identified in these specimens.

### Dentinal tubules occlusion by the tested formulations

**Figure 4:**
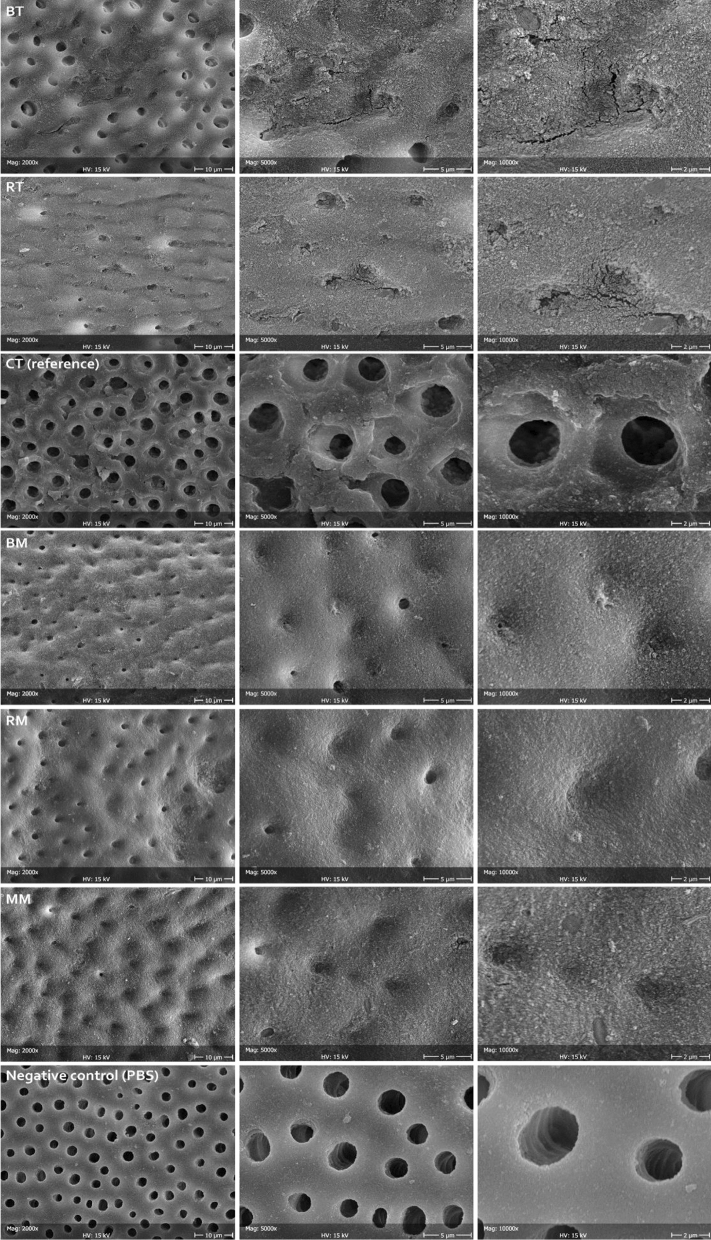
Representative micrographs at different magnifications (2000 × , 5000 × , and 10,000 ×) of the dentine surfaces after treatment with test and reference formulations, compared to the negative control (demineralized surface stored in PBS). Open tubules can be clearly seen that were closed in a variable percentage depending on the tested formulation. In general, mousses provided better obliteration (increased layer homogeneity) of the tubules than the toothpastes.

The negative control (PBS) showed that all tubules exposed by the demineralization procedure remained open. The reference treatment (CT) showed the occlusion of very few tubules. In contrast, all tested topical formulations showed complete occlusion of the dentinal tubules, while the toothpastes could occlude the majority of the exposed tubules (Fig. 4). An amorphous mineral layer could be identified on the surface of the specimens, occluding the tubules opening.

**Figure 5:**
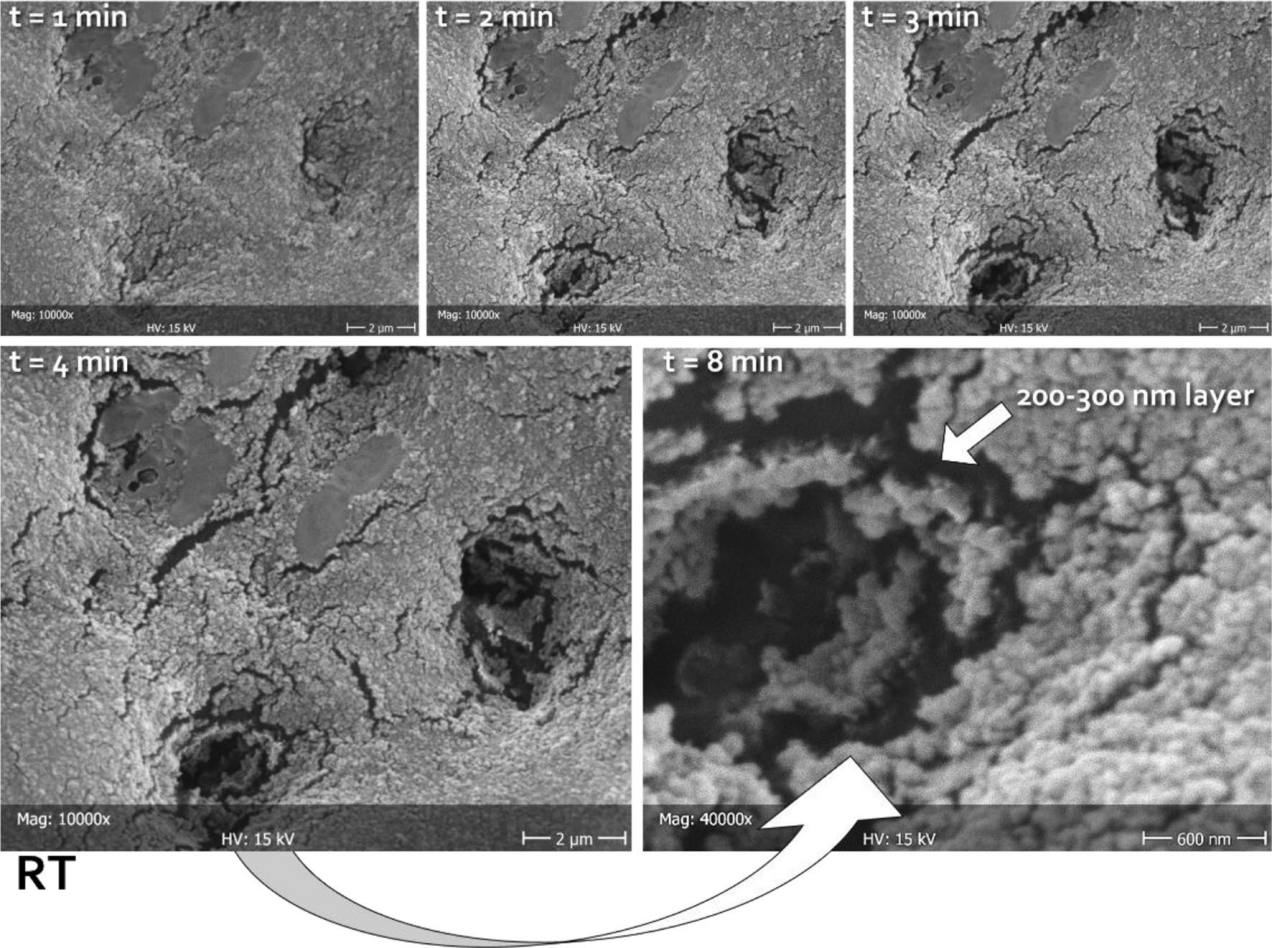
Representative micrographs of the same area (in this case belonging to RT), scanned at different times (1, 2, 3, 4, and 8 min) focusing the electron beam on the opening of tubules, thus heating the carbon-rich collagen structures underneath and disrupting the mineralized material obliterating the lumen. In this way, it was possible to measure the thickness of the deposited layer. The latter can be clearly seen as constituted by a homogeneous assembly of spherical nanoparticles. The absence of exposed HA prisms in this substratum did not allow for epitaxial growth; therefore, no HA nanocrystals could be identified independently from the tested formulations. Considering the treatment time that was allowed in this study (one week), it can be speculated that the tested formulations might need to incorporate a scaffold in order to be able to grow organized structures on the dentinal substrate.

To measure the thickness of the deposited layer, it was beamed with the electron gun while sequentially focusing it on the opening of single tubules. It was observed that, due to its reduced thickness, the layer collapsed after few minutes, exposing the tubule lumen (Fig. 5). It is likely that carbon-rich collagen structures underneath the tubule opening were denatured by heating with the electron gun, thus collapsing. Therefore, it was possible to show that the layer had a thickness of about 200 nm in all remineralizing formulations. Beaming the layer that was deposited on the intertubular dentine produced no effect.

### Elemental surface analysis of treated enamel specimens

The results of EDS analysis performed on enamel specimens treated with the different formulations to study their surface elemental composition is presented in Table 5. The enamel negative control specimens showed the presence of C, Ca, P, Na, Mg, and Cl. The presence of Si traces can be explained by the polishing procedures performed, despite thorough cleaning and acid treatment. The Ca/P molar ratio was 1.91 ± 0.02, which is typical of enamel HA. Specimens treated with BT, RT, CT, and MM showed a similar Ca/P ratio (1.91–1.95 ± 0.05).

**Table 5:** Energy-dispersive X-ray spectroscopy (EDS) compositional analysis of enamel surface layer (≈1 μm) brushed with the tested formulations.

wt%	BT	RT	CT (reference)	BM	RM	MM	Negative control (PBS)
C	6.23(1.13)^b^	6.57(0.60)^a,b^	6.88(0.95)^a,b^	6.47(0.13)^a,b^	6.77(1.44)^a,b^	7.24(0.64)^a^	7.52(0.78)^a^
O	40.08(0.42)^a,b^	39.76(0.67)^a,b^	39.37(1,24)^b^	41.50(0.51)^a^	41.48(0.34)^a^	39.22(1.10)^b^	40,27(0,92)^a^
P	14.7(0.39)^a^	14.8(0.53)^a^	14.81(0.59)^a^	13.41(0.84)^b^	13.70(0.44)^b^	14.54(0.19)^a^	14.56(0.38)^a^
Ca	37.09(0.67)^a^	36.58(0.58)^a,b^	36.50(1.14)^a,b^	35.40(1.52)^b^	35.59(0.57)^b^	36.50(0.70)^a,b^	35.92(0.92)^b^
F	0.19(0.06)^b^	0.05(0.04)^d^	0.11(0.05)^c^	0.28(0.07)^a^	0.01(0.01)^d^	0.20(0.03)^b^	0.03(0.02)^d^
Mg	0.27(0.08)^a^	0.21(0.05)^b^	0.22(0.06)^a,b^	0.17(0.04)^b^	0.20(0.01)^a,b^	0.23(0.01)^a,b^	0.21(0.02)^a,b^
Zn	0.07(0.09)^c^	0.23(0.06)^a^	0.10(0.07)^b,c^	0.04(0.06)^c^	0.22(0.02)^a,b^	0.03(0.06)^c^	0.09(0.08)^c^
Sr	0.34(0.04)^a^	0.10(0.13)^c^	0.23(0.04)^b^	0.38(0.08)^a^	0.23(0.04)^b^	0.21(0.04)^b^	0.22(0.10)^b^
Na	0.48(0.17)^a^	0.63(0.24)^a^	0.64(0.23)^a^	0.57(0.28)^a^	0.56(0.03)^a^	0.55(0.04)^a^	0.60(0.06)^a^
Al	0.01(0.01)^b^	0.00(0.00)^b^	0.01(0.01)^b^	0.02(0.01)^a,b^	0.00(0.00)^b^	0.00(0.00)^b^	0.03(0.03)^a^
Si	0.30(0.12)^c^	0.73(0.27)^b^	0.73(0.28)^b^	1.66(0.15)^a^	1.08(0.38)^b^	0.68(0.08)^b^	0.21(0.05)^c^
S	0.00(0.00)^b^	0.09(0.10)^a^	0.01(0.02)^b^	0.00(0.00)^b^	0.02(0.03)^a,b^	0.07(0.08)^a^	0.02(0.03)^b^
Cl	0.18(0.06)^b,c^	0.19(0.08)^b,c^	0.22(0.07)^a,b^	0.10(0.04)^c^	0.15(0.05)^b,c^	0.26(0.03)^a^	0.26(0.06)^a^
K	0.06(0.06)^a^	0.05(0.07)^a^	0.03(0.05)^a^	0.02(0.01)^a^	0.00(0.00)^a^	0.01(0.02)^a^	0.05(0.07)^a^
Ti	0.00(0.00)^b^	0.00(0.01)^b^	0.14(0.03)^a^	0.01(0.01)^b^	0.01(0.01)^b^	0.29(0.05)^a^	0.00(0.01)^b^
Ca/P (mol)	1.95(0.05)^a,b^	1.91(0.06)^b^	1.91(0.05)^b^	2.05(0.22)^a^	2.01(0.03)^a,b^	1.94(0.02)^a,b^	1.91(0.02)^b^

Means ± 1 standard deviation were calculated from normalized wt% data. Different superscript letters indicate, for each element, significant differences between formulation treatments effect on the enamel surfaces (Tukey’s test, p < 0.05).

Traces of F and Sr were detected on BT- and BM-treated surfaces, while Zn was detected on RT- and RM-treated ones. The increased presence of Si on RT, RM, BM, and MM-treated surfaces compared to the negative control and the presence of F, Sr, and Zn indicated that a new mineral phase had been deposited on eroded enamel. This new mineral phase had a similar chemical composition to biogenic apatite (including the degree of carbonated HA) enriched with the doping ions and containing traces of silica derived from the toothpastes. The tested mousses deposited a surface layer with a relatively higher Ca/P molar ratio (2.05–2.01 ± 0.03). F and Si content of the layer formed after BM treatment was significantly higher compared to BT. The other doping ions were present in similar amounts to the toothpastes.

The specimens treated with the reference toothpaste showed significant amounts of F and Ti deposition. In particular, F presence was significantly lower than specimens treated with BT and mousse.

## Discussion

The present study structurally characterized and compared the remineralizing effect and tubules occlusion capabilities of different commercially available toothpastes and topical formulations. It also associated the composition of the tested formulation with their activity. Every formulation had its unique composition, varying in terms of calcium phosphate bioactive nanocompounds, calcium phosphates to silica ratio, and ion doping. Nevertheless, all tested formulations containing bioactive nanocompounds showed similar remineralizing effects thanks to the deposition of biomimetic, ion-substituted HA (chemically similar to biogenic HA) on demineralized enamel and exposed dentinal tubules. The null hypothesis can thus be rejected. The fact that all tested bioactive formulations achieved significant and similar remineralization capabilities is quite surprising and possibly means that doped nanohydroxyapatite and metastable ACP compounds are equally effective. Also, under the tested conditions, the mousse formulations were no better performers than toothpastes, meaning that differences in clinical outcomes may be due to different application procedures (wearing a tray vs. toothbrushing) rather than differences in the formulation. At the bottom line, enamel remineralization was likely promoted by the supply of Ca and P in the needed ratio and amounts, by regulatory elements such as the doping ions or Cit-ACP and CPP-ACP, and by the presence of additional nucleation sites provided by silica nanoparticles. Since these factors were all present in the tested formulations, they could display remineralization capabilities. Tschoppe et al. tested different nano-HA toothpastes, obtaining similar remineralizing capacities with enamel and dentine lesions obtained *in vitro*^22^. For dentine, higher remineralization effects were achieved using nano-HA or Zn^2+^, CO_3_^2−^ doped nano-HA toothpastes compared to a control amine fluoride toothpaste. In spite of differences in the methodology applied, the study confirms our results and the increasing importance of remineralization strategies based on ion-doped Ca-P bioactive nanocompounds.

Structural characterization generally showed good correlation with the declared compositions. PXRD, FTIR-ATR, and chemical characterizations confirmed that the inorganic water-insoluble fraction of the toothpaste formulations were composed of amorphous silica and HA. Results showed that, where present, HA was amorphous and doped with CO_3_^2−^ and either Zn^2+^ or F^−^, Mg^2+^, and Sr^2+^ ions. PXRD and FT-IR patterns relative to amorphous SiO_2_ content and HA, where detected, are well in keeping with the literature data^23–27^. The HA peaks of both BT and RT toothpastes suggest a high similarity to biogenic HA^28^. This finding can explain the morphological observations, where epitaxial growth of a new HA crystallites layer can be seen in continuity with the pristine enamel structures, without any interruptions.

According to the manufacturer’s specifications, BM is a water-free product. The water content of the tested formulations was calculated as weight difference after freeze-drying. Therefore, the water content detected in BM might be due to moisture and volatile components. However, none of its main contents (glycerol, PEG-8) are volatile under the tested experimental conditions applied (freeze-drying). In contrast, both substances are highly hygroscopic, which explains their presence in BM formulation to prevent ACP conversion to HA. Moisture incorporation may therefore seem the main reason explaining water presence. The aliquots were immediately tested as soon as dispensed, ruling out the incorporation of significant amounts of moisture from the test laboratory room. Also, PXRD showed relatively low and broad HA peaks, the finding being confirmed by FT-IR analysis. This result may come as a surprise, since BM does not contain HA, rather than calcium phosphate carbonate citrate fluoride as per manufacturer’s specifications, that is, Cit-ACP. There is a general agreement that ACP crystallizes and converts into HA in the presence of water^8^. Therefore, the presence of HA in the can be due to Cit-ACP crystallization during the washing process, being thus considered a consequence of the applied characterization procedures. On the contrary, MM contains water in its composition, so one could speculate that the identification of HA in its water-insoluble fraction may be due to a time-dependent conversion of the ACP to HA. However, ACP comes complexed as CPP-ACP^21^, and significant conversion to HA is unlikely within the shelf life of the formulation. The applied washing process is therefore responsible once again for the crystallization of the ACP contained in the MM formulation.

The compositional analyses focused on the presence of Mg^2+^, Sr^2+^, Zn^2+^, and F^-^ ions since, according to the literature, they are involved in remineralizing^29,30^ and antibacterial activity^31,32^. Analyses were generally in good correlation with one another and with the declared content. Since all tested formulations showed remineralizing activity, it can be assumed that the presence of such ions as well as of CPP helps in modulating such activity. In particular, Mg^2+^ adsorbed on the surface of amorphous calcium phosphate retard the transformation to HA^33^. Such ion is present in native HA and plays an important role, being able to influence the type of crystal that is formed^34^. Sr^2+^, similarly to Mg^2+^, substitutes for Ca^2+^ but instead of inhibiting crystal growth, it increases lattice parameters, while not having any influence on crystallinity and thermostability of the formed HA^35^. Apart from the well-known antimicrobial effect and increased resistance to demineralization of fluoride ions^15–17^, it has been shown that the addition of F^-^ speeds up the conversion of F-ACP to F-HA, that, in turn, tends to adhere to highly crystalline prismatic regions^8^. The effect of HA doping with Zn^2+^ ions is the formation of precipitated crystals with reduced length and width. Such differences may explain the morphological findings that showed, after the tested incubation time, a more pronounced nanocrystallite deposition when HA was doped with Mg^2+^, Sr^2+^, and F^−^ rather than Zn^2+^. For this reason, the doping with the aforementioned ions seems the most promising approach among the tested ones in achieving enamel remineralization.

It must also be mentioned that, whenever present, HA was carbonated. HA doping with CO_3_^2−^ that have the primary effect of increasing its solubility and, therefore, its reactivity. Finally, all tested formulations contained SiO_2_. The latter compound is added to most toothpaste formulations to provide abrasiveness. However, silicates have been proven to be stronger inducers of remineralization even than fluoride, calcium or phosphate^36^. The spherical nanoclusters that were found in the present study in-between newly formed HA crystals can be identified as silica that may play an important role, still to be fully clarified, in HA deposition. One may speculate that flat surfaces of enamel prisms act as nucleation barriers. Inert silica nanoparticles that provide nucleation centers could help in overcoming such barriers.

All topical formulations showed a better tubules occlusion compared to the toothpastes. This finding can be explained by a higher concentration of Ca-P bioactive nanocompounds. An exception was MM that contained surprisingly low amounts of Ca and P. The latter finding can be explained by the bioactive compound present as CPP-ACP. The casein phosphopeptide is a nanocomplex acting as a reservoir for calcium and phosphate ions^37^, thus explaining, at the same time, a relatively high carbon presence (peptide chain) and a relatively low Ca and P presence in the tested formulation.

In contrast with enamel remineralization, the deposited layer that occluded dentinal tubules was mainly made by spherical nanoparticles rather than HA nanocrystallites. This datum suggests the need for additional scaffolding such as self-assembly peptides, or poly ascorbic acid to achieve epitaxial growth and improve bioremineralization processes, particularly on the dentine substrate.

A direct correlation between the diameter/density of open dentinal tubules and teeth hypersensitivity is well reported in the literature^38,39^. Although the data presented in this work are in vitro results, it can be speculated that the high degree of dentinal tubules occlusion exerted by the tested formulations could lead to a desensitizing clinical effect. Additional investigations need to be carried out to clinically study the effect of the formulations on enamel and dentin substrates in the long term.

**Figure 6:**
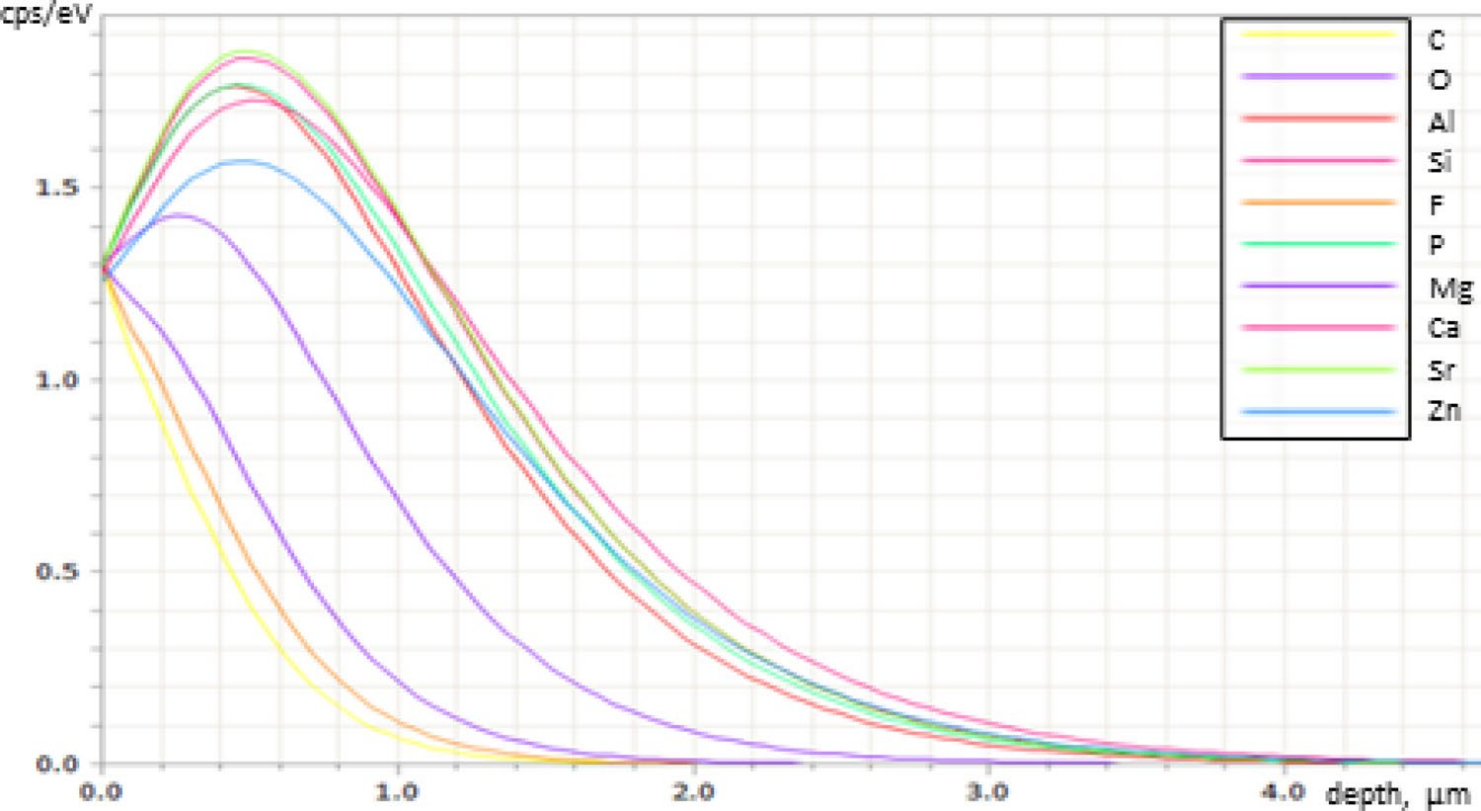
Energy-dispersive X-ray spectroscopy (EDS) spectra of depth interaction at 15 kV electron accelerating voltage depending on the different elements. While carbon, oxygen, and fluoride signals originate from a very superficial layer (most of the signal comes from the first 600 nm), the information of all other elements comes from a much thicker layer where most information comes from the first 1200 nm. This distribution has to be kept in mind when analyzing the elemental compositions of the remineralized layers having a thickness of 200 to 300 nm (Fig. 5).

From the point of view of the analytic technique, EDS provided a complete elemental profile of the tested formulations, even when elements were present in traces (less than ≈1 wt%). It has to be kept in mind that the observation was performed on the outer part of a dry mass, whose composition might be slightly different from its bulk. Also, the acquired data represented the elemental composition of the ≈1 μm superficial layer. Since the thickness of the observed remineralization layer deposited on dentine was found to be 200 to 300 nm independently of the tested formulation (Fig. 5), it can be inferred that the signal provided by the EDS probe is composed of information coming from both the remineralized layer and the underlying substrate to a varying amount, depending on the atomic number of the assessed element (Fig. 6). Therefore, the absolute values of elemental composition, including the calculated Ca/P molar ratios, despite having good correspondence to enamel HA^40,41^, have to be interpreted with the necessary caution. Indeed, the analysis was performed to confirm that the remineralized layer originated from the tested formulation whenever observed on the specimens’ surface.

In the present study, SEM electron beaming was used to evaluate the thickness of the observed remineralization layer deposited on dentine. Conventional approach include transverse microradiography that can provide useful information on the subsurface lesion characteristics^42^. However, a drawback of such analysis is the difficulty that is often encountered in obtaining thin sections of small and brittle specimens, that can often lead to specimen loss^22^. The methodology used in the present study, while far from being comprehensive, allows nevertheless for a much lower invasiveness in the determination of newly deposited layers that may be difficult to be visualized using other techniques for specimen processing. An improvement of such method that may combine the advantages of transverse microradiography and electron beaming would be focused ion beam sectioning coupled with SEM imaging (FIB-SEM). Also, nanoindentation would provide useful information about the newly deposited layer^42^.

## Conclusions

In contrast with a conventional fluoride-containing toothpaste, all formulations containing Ca-P bioactive nanocompounds showed remineralizing ability by epitaxial growth of a layer showing the morphology and composition of human hydroxyapatite. The presence of doping ions (especially CO_3_^2−^, F^−^, Mg^2+^, and Sr^2+^) or casein phosphopeptide seemed essential to allow such performances. Topical mousse formulations showed a higher tubules occlusion capability than toothpastes, independently from their composition. These results suggest the market-availability and widespreading of a technology that was only available in the lab until recently. This study can be situated in the frame of the possibility to restore tooth structures in a biomimetic way, contrasting dental demineralization processes leading to caries.

## Methods

### Materials

The tested toothpastes and topical formulations and their composition as specified by the manufacturer are presented in Table 1. Modified Dulbecco’s phosphate-buffered saline (PBS, without CaCl_2_ and MgCl_2_), orthophosphoric acid (H_3_PO_4_ 85 wt.% in H_2_O), hydrochloric acid (HCl 37 wt.% in H_2_O), and hydrofluoric acid (HF 40 wt.% in H_2_O) were purchased from Sigma Aldrich (St. Louis, MO, USA). All the solutions were prepared with ultrapure water as well as washing procedures (18.2 MΩ × cm, 25 °C, Arium pro, Sartorius, Göttingen, Germany).

### Sample preparation

An aliquot of each toothpaste was freeze-dried overnight at − 50 °C under a vacuum of 3 mbar and then weighted to estimate its water content. Each toothpaste was then washed with ultrapure water to separate the water-insoluble fraction. A total of 5 g of each toothpaste was dispersed in 150 mL of ultrapure water. Then, three repeated rinses with water were performed, each followed by centrifugation at 10,000 rpm for 5 min. The compounds were freeze-dried overnight, ground, and sieved with a 50 μm sieve to achieve uniform granulometry. The resulting dried powders were weighted to quantify the water-insoluble fraction of the toothpaste. Then, an aliquot was dissolved in a 6 wt.% HNO_3_ solution to collect the acid-insoluble fraction. The latter fraction was collected by centrifugation (10,000 rpm for 5 min), freeze-dried overnight, and then weighted^18^.

### Structural characterization

Powder X-ray diffraction (PXRD) patterns of the water-insoluble and acid-insoluble fractions of the products were measured in the angular region from 10 to 60° (2θ) with a step size of 0.02° and a collection time of 0.5 s. Cu Kα X-rays (λ = 1.5418 Å) were generated at 40 kV and 40 mA. The PXRD patterns were collected on a D8 Advance diffractometer (Bruker, Karlsruhe, Germany) equipped with a Lynx-eye detector.

Fourier transform infrared (FT-IR) spectra of the freeze-dried products, the water-insoluble fractions, and the acid-insoluble fractions of the products were collected with a Nicolet iS5 instrument (Thermo Fisher Scientific Inc., Waltham, MA, USA) equipped with iD7 attenuated total reflectance (ATR) accessory.

### Compositional analysis

Quantification of Mg^2+^, Sr^2+^, and Zn^2+^ in the whole products and their water-insoluble fractions was performed with an inductively-coupled plasma optical emission spectrometer (ICP-OES, Agilent 5100, Agilent Technologies, Santa Clara, CA, USA). Samples were prepared by dissolving 100 mg of the whole product or water-insoluble fraction in 50 mL of a 0.8 wt.% HF solution.

The quantification of F^-^ in the whole formulations was performed with a fluoride ion-selective electrode (ISE, Intellical ISEF121, Hach Lange, Loveland, CO, USA). A total of 6 mL of an aqueous dispersion of the product (0.1 g mL^−1^) was mixed with an equal volume of 37 wt.% HCl and left to react for 1 h at 50 °C to dissociate fluoride ions completely^43^. Afterward, the acid-insoluble residue was removed by centrifugation (14,000 rpm, 3 min), and the supernatant was analyzed using the protocol suggested by the instrument’s manufacturer.

An aliquot of the whole formulation (1 g) was also dried overnight in the vacuum bell, then in the observation chamber of a tabletop SEM, and observed using energy-dispersive X-ray spectroscopy (EDS). The analysis was performed using a tabletop scanning electron microscope (TM4000Plus, Hitachi, Schaumburg, IL, USA) equipped with an EDS probe (Q75, Bruker, Berlin, Germany). Specimens were observed in surface-charge reduction mode without sputter-coating, using an accelerating voltage of 15 kV. Three randomly selected fields were acquired for each specimen (three aliquots per each formulation from three different tubes) at 300 × magnification in full-frame mode using an acquisition time of 150 s^44^. Acquired data represented the elemental composition of the ≈1 μm superficial layer (depth of sampling depends on the atomic number of sampled elements, see Fig. 6).

### In vitro evaluation of remineralization and dentinal tubules occlusion

A total of 18 sound (caries-free) erupted human wisdom teeth extracted for clinical reasons were obtained and used for the in vitro experiments (Oral Surgery Unit, Department of Biomedical, Surgical and Dental Sciences, Milan, Italy). The Ethical Commission of the University of Milan approved the use of the teeth (ethical vote number SALTiBO–2017, date of approval, July 11, 2017). Each donor signed informed consent in a written form. All in vitro experiments were performed in accordance with the Declaration of Helsinki, updated by the World Medical Association in 2013.

**Figure 7:**
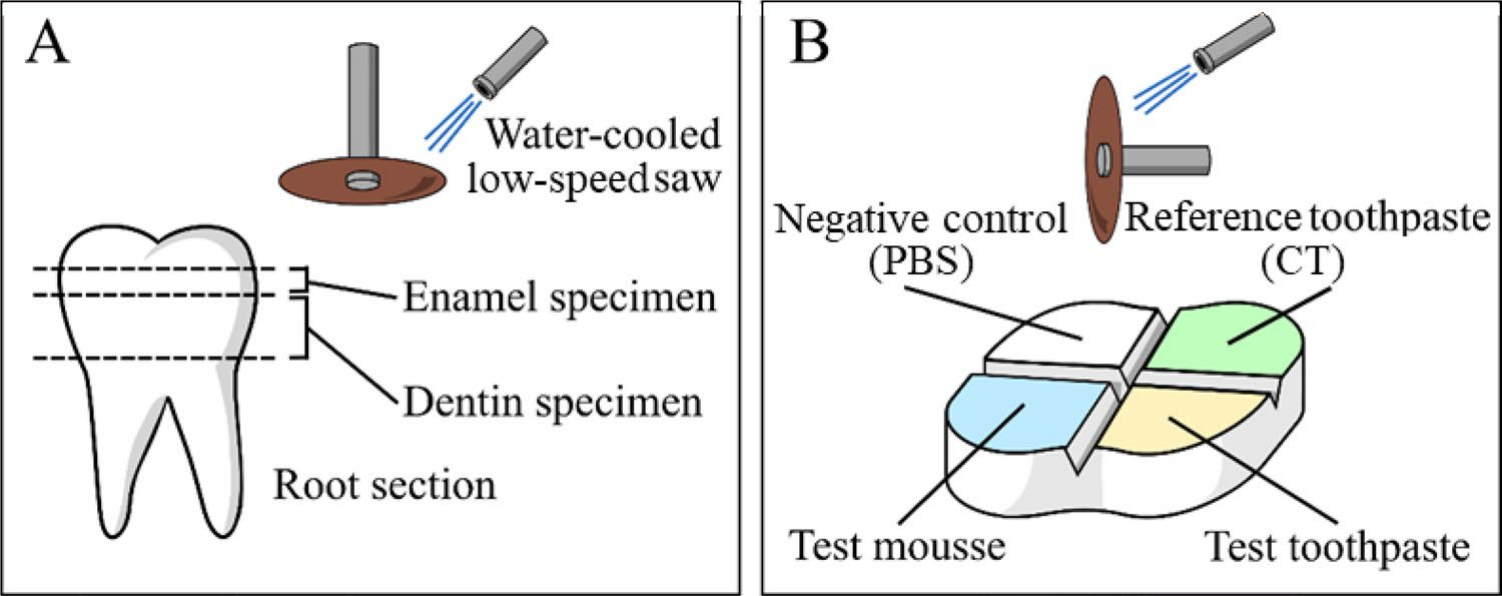
Diagrams depicting specimen preparation. (**A**) horizontal sections of the tooth exposing enamel and dentin and (**B**) delimitation of control and treatment regions by notches made using the diamond disc. In all enamel and dentine specimens (n = 18 each), one of the 4 delimited regions was always kept as the negative control, leaving the other three regions randomly allocated to one of the six toothpastes or mousse treatments (9 regions per each treatment, 18 regions for the negative controls). The negative control regions were used to ensure homogeneous structures among specimens (enamel demineralization pattern, dentine tubules density).

Horizontal sections of the teeth specimens were obtained under constant water cooling using a low-speed saw (Isomet 1000, Buehler, Lake Bluff, IL, USA). Cuts were performed at two depth levels in order to section the root 2 mm apical to the cementoenamel junction and to obtain flat enamel (n = 18) or dentin (n = 18) surfaces (Fig. 7A). Then, on both dentine and enamel sections, two perpendicular 0.5 mm-deep notches were made on the top surface of each specimen to mark four areas using a low-speed diamond disc (Horico, Berlin, Germany, Fig. 7B). All surfaces were polished using silicon carbide paper (600 and 1200 grit), and the surfaces were etched with a 37 wt.% H_3_PO_4_ solution for 30 s, followed by extensive rinsing with water^18^.

The toothpastes were soft-brushed for 3 min using disposable microbrushes (Microbrush, Microbrush International, Grafton, WI, USA), then they were rinsed with ultrapure water for 1 min. Each toothpaste was applied on a single region of a specimen section, either enamel (n = 9 regions) or dentine (n = 9 regions), to test enamel remineralization and dentinal tubule occlusion, respectively (Fig. 7). This treatment was repeated three times each day for one week. Specimens were stored in fresh PBS at 37 °C between treatments.

After the last treatment, all specimens were left to dry overnight in a desiccator. Then, conductive tape was used to fix them on stubs before storage in a vacuum bell for five hours. After that, they were sputter-coated with gold (Polaron Sputter Coater E5100, Polaron Equipment, Watford, UK) and observed with an SEM (JSM 840A, JEOL, Tokyo, Japan) in secondary electrons mode at an acceleration voltage of 10 kV. For each specimen and toothpaste treatment, four randomly selected fields were acquired at magnifications ranging from 500 × to 40,000 × . The enamel remineralization and dentinal tubule occlusion ability of the tested formulations were compared to a conventional fluoride-containing toothpaste (reference CT, Table 1) that is considered a standard treatment in the prevention of demineralization.

### Elemental composition of the specimen surface

EDS analysis was performed as previously specified on treated specimens after vacuum-drying but before sputter-coating. Surface-charge reduction mode and an accelerating voltage of 15 kV were used. Three randomly selected fields were acquired for each enamel specimen and treatment at 300 × magnification.

### Statistical analysis

Structural characterization and compositional analysis experiments were performed in triplicate and repeated at least three times. Such data are displayed in the manuscript as means of the three experiments ± 1 standard deviation. EDS data were analyzed using statistical software (JMP 10.0, SAS Institute, Cary, NC, USA) to compare the presence of each element between the toothpaste treatments. ANOVA and Tukey’s HSD post-hoc test were applied setting the significance level to p < 0.05, prior verification of normality of distribution and homoscedasticity was performed by Shapiro Wilk’s and Levène’s tests, respectively (p < 0.05).
